# The Impact of Vitamin D in the Prevention of Influenza, COVID-19, and Dengue: A Review

**DOI:** 10.3390/biomedicines13040927

**Published:** 2025-04-09

**Authors:** Mario Galindo-Méndez, Mario Galindo-Ruiz, María Florencia Concheso-Venegas, Sebastián Uriel Mendoza-Molina, David Orozco-Cruz, Efraín Weintraub-Benzion

**Affiliations:** 1 Laboratorios Galindo SC, Av Juárez 501-A, Oaxaca, Oaxaca CP 68000, Mexico; mario.galindoru@anahuac.mx; 2 Escuela de Medicina, Universidad Anáhuac Oaxaca, Blvd. Guadalupe Hinojosa de Murat 1100, San Raymundo Jalpan, Oaxaca CP 71248, Mexico; sebastian.mendozam@anahuac.mx (S.U.M.-M.); david.orozco@anahuac.mx (D.O.-C.); 3 Escuela de Medicina, Universidad Anáhuac Campus Norte, Av. Universidad Anáhuac 46, Huixquilucan, Estado de Mexico CP 52786, Mexico; maria.conchesove@anahuac.mx (M.F.C.-V.); efrain.weintraubbe@anahuac.mx (E.W.-B.)

**Keywords:** vitamin D, dengue, COVID-19, influenza, inflammatory response, immune modulation, acute respiratory distress syndrome

## Abstract

Since its discovery, vitamin D (VD) has been known for its implications in maintaining bone homeostasis. However, in recent years it has been discovered that the vitamin D receptor is expressed on different cells of the immune system and that these cells can locally produce the active form of this molecule, calcitriol, strongly suggesting that this vitamin might play a key role in both branches of the immune system, innate and adaptive. Recent evidence has demonstrated that VD participates in the different protective phases of the immune system against invading microorganisms, including in the activation and production of antimicrobial peptides, in the inactivation of replication of infectious agents, in the prevention of the exposure of cellular receptors to microbial adhesion, and, more importantly, in the modulation of the inflammatory response. In recent years, the world has witnessed major outbreaks of an ancient infectious disease, dengue fever; the emergence of a pandemic caused by an unknown virus, SARS-CoV-2; and the resurgence of a common respiratory infection, influenza. Despite belonging to different viral families, the etiological agents of these infections present a common trait: their capacity to cause complications not only through their cytopathic effect on target tissues but also through the excessive inflammatory response produced by the human host against an infection. This review outlines the current understanding of the role that vitamin D plays in the prevention of the aforementioned diseases and in the development of their complications through its active participation as a major modulator of the immune response.

## 1. Introduction

Vitamin D molecules have been present in organisms for at least 1.2 billion years, ever since eukaryotic cells were first able to synthesize sterols and, thus, produce these molecules when exposed to the ultraviolet rays of sunlight [1]. Rickets disease in children and osteomalacia in adults are pathologies that were first described centuries ago, with the former being considered the oldest-known pathology of a vitamin deficiency [2]. However, it was not until 1922, with the studies performed by Elmer McCollum on dogs and rats, that the molecule responsible for curing rickets and regulating bone metabolism was discovered and found to be a previously unknown vitamin [3]. Since this new molecule was the fourth vitamin to be discovered, it received the name vitamin D (VD). 

Since the discovery of vitamin D, it has been known that this molecule plays a key role in the homeostasis of phosphorus and calcium. However, since the discovery that the vitamin D receptor (VDR) is expressed by different cell lines (e.g., keratinocytes, fibroblasts, pancreatic and lung cells), in addition to bone and intestinal cells, and that the enzyme 1α-hydroxylase, responsible for the conversion of pre-vitamin D to its active form, is also expressed by different tissues, independent studies have related VD deficiency to extra-skeletal pathologies. Among these “non-classical” entities related to the scarcity of this molecule are obesity, diabetes, autoimmune and cardiac diseases, and cancer. Nevertheless, since the relationship between hypovitaminosis D and these diseases has not been conclusively demonstrated, this deficiency could only be a consequence and not the root cause of these diseases; therefore, the possible association between vitamin D deficiency and these pathologies must be carefully considered [4,5]. In addition to the abovementioned pathologies, it has been identified that vitamin D directly impacts the regulation of different cells and molecules of the immune response [6,7,8].

The first evidence of a possible relationship between vitamin D and the immune system emerged in the mid-19th century with the research of CJB Williams, who showed that the majority of his patients infected with tuberculosis presented a “great improvement” after treatment with cod liver oil [9]. In recent years, after it was found that different cells of the immune system express the vitamin D receptor and that these cells respond to the action of calcitriol [6,7], it has been confirmed that VD is a potent regulator of the innate and the acquired immune responses.

In recent years, research has shown that the VD-VDR complex regulates the expression of genes involved in the production of antimicrobial peptides (cathelicidins, defensins, hepcidins) [10], actively participates in the differentiation of antigen-presenting cells [11], influences the development of dendritic cells with tolerogenic properties, as well as the antigen presentation by these cells [9], and inhibits the expression of major histocompatibility complex II molecules [11]. Among the adaptive branch processes of the immune system where vitamin D has been described as a major participant are the activation of cell-mediated (Th1) and humoral-mediated (Th2) immunity [12], the regulation of the expression of both pro- and anti-inflammatory interleukins [13], and the inhibition of the differentiation and proliferation of antibody-producing B cells [14].

Due to the impact of vitamin D on the immune response, different authors have suggested a possible relationship between a deficiency in this prohormone and the development of certain infectious diseases, or have proposed treatment based on this vitamin as a preventive measure for complications related to these infections. Examples of infectious diseases in which vitamin D treatment could positively affect the outcomes include hepatitis C [15], tuberculosis [16], HIV/AIDS [17], and dengue [18], as well as respiratory infections such as COVID-19 [19] and influenza [20].

In the last few years, Mexico has experienced major outbreaks of dengue, with over 125,000 cases reported in the year 2024 alone [21]. In addition, in our country, as in the rest of the world, numerous cases of infections due to SARS-CoV-2 and the influenza virus have been reported. Although these infections are caused by three very different viruses, there are striking similarities in their pathologies: a hyperinflammatory response, mediated by a cytokine storm and characterized by the overproduction of interleukin 6 (IL-6) and 8 (IL-8) and delayed and diminished interferon gamma (IFN-γ) expression [22]. The objective of the current review was to present evidence of the role played by vitamin D in the prevention of COVID-19, influenza and dengue, as well as in the prevention of complications related to these infections, and to present the possible future research that can contribute to obtaining greater knowledge about the relationship between vitamin D and the immune system.

## 2. Metabolism and Mechanism of Action of Vitamin D

In nature, vitamin D is found in two forms: ergocalciferol, also known as vitamin D2 (VD2), and cholecalciferol, also known as vitamin D3 (VD3). In humans, most of the circulating VD is produced by a series of chemical reactions that begin in the cellular membranes of the dermis and the epidermis, where the 7-dehydrocholesterol molecule is transformed into pre-cholecalciferol (pre-vitamin D3) by the action of ultraviolet radiation from the sun [23]. Due to the thermal instability of this new compound at body temperature, pre-cholecalciferol is converted into a stable molecule, cholecalciferol (VD3). As an autoregulatory mechanism of the human body, the continuous radiation of pre-vitamin D leads to the reversible formation of other compounds, such as lumisterol 3 and tachysterol 3, both of which can revert to pre-vitamin D in the dark [24].

In addition to the cutaneous synthesis of vitamin D, this vitamin can also be acquired through the ingestion of foods of animal origin (cholecalciferol) or plant origin (ergocalciferol). According to the Dietary Guidelines for Americans (DGAC), some of the food products that contain this vitamin are fish such as salmon, sardines, and tilapia, as well as eggs, dairy products, and mushrooms [25].

**Figure 1:**
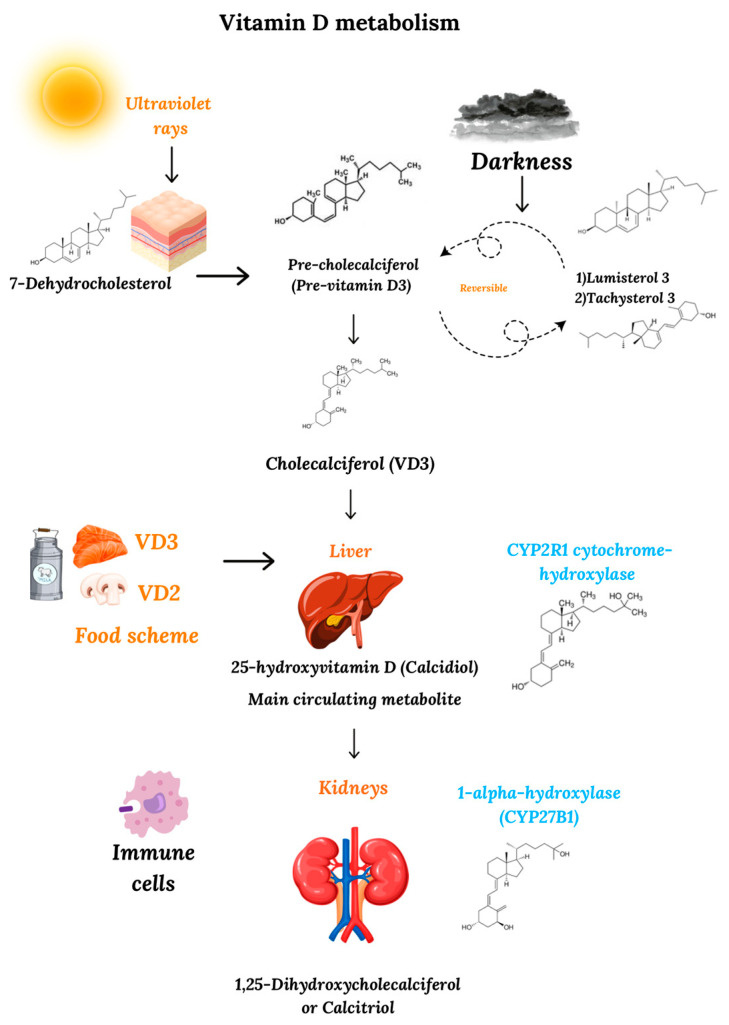
Metabolism of vitamin D. The synthesis of VD involves different cells of the body, beginning at the dermis and epidermis, where 7-dehydrocholesterol is transformed into pre-vitamin D and, after a series of chemical reactions in the liver and kidneys, is converted into its active form, calcitriol. Additional cells participating in the synthesis of VD are those of the immune system, which express 1α-hydroxylase and the VD receptor, being able to locally produce calcitriol.

Regardless of the mechanism of its synthesis or origin, a vitamin D molecule requires two hydroxylation reactions to become biologically active. The first of these reactions primarily takes place in the liver, after vitamin D is transported to this organ by the vitamin D-binding protein (DBP) and, to a lesser extent, albumin [26]. In the liver, VD is hydroxylated at position 25 in VD3 and position 24 in VD2 [26], mainly by the CYP2R1 cytochrome hydroxylase, a highly specific enzyme for vitamins D3 and D2, transforming both molecules into 25-hydroxyvitamin D or calcidiol (25(OH)D). Literature data indicate that CYP2R1 might not be the only VD-hydroxylase, but is the most critical in humans, as genetic variations in this enzyme, but not in other cytochrome hydroxylases, are responsible for serum 25(OH)D deficiency [27]. The second enzymatic reaction for the activation of VD takes place primarily in the kidney, due to the action of the 1α-hydroxylase enzyme (CYP27B1) on position one of calcidiol, converting 25(OH)D into the biologically active form of VD, 1,25-(OH)2 vitamin D or calcitriol [28] (Figure 1). Despite the importance of renal 1α-hydroxylase in the activation of VD, research has shown that this enzyme is also expressed by extra-renal cell lines, such as the skin, colon, pancreas, brain, and placenta cells, indicating the active participation of these tissues in the extra-renal activation of vitamin D [29]. In addition to these cell lines, CYP27B1 is also expressed by immune cells and is thought to modulate immune function in a manner similar to cytokines. Whereas the expression of 1α-hydroxylase by the kidneys is primarily mediated by parathyroid hormone and fibroblast growth factor 23 (FGF23) [30], which is released in response to low serum calcium levels and acts predominantly as an endocrine hormone, CYP27B1 expression is triggered in immune cells by cytokines such as IFN-γ or by microbial molecule signaling through Toll-like receptors such as TLR3 and TLR4 [31], acting in an intracrine or paracrine manner. Thus, in VD production by immune cells, this prohormone is produced locally to respond to infections and represents a key role of calcitriol in immune system regulation.

**Figure 2:**
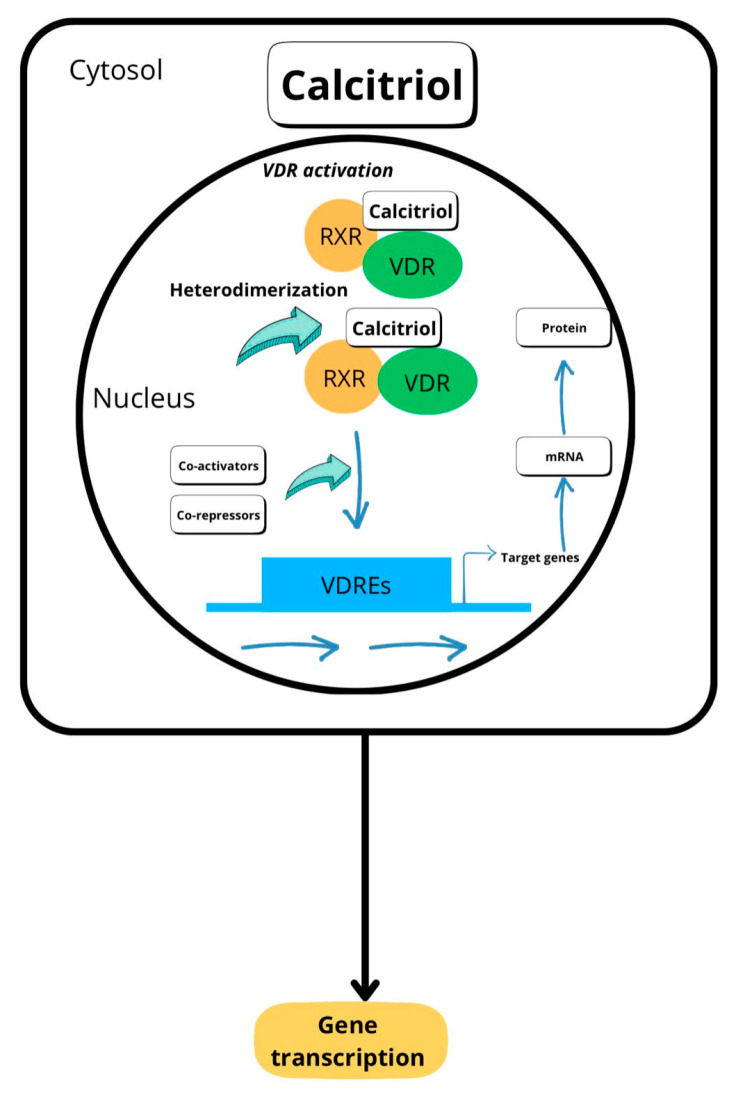
Vitamin D mechanism of action. After cell internalization, VD binds its receptor (VDR) and one of the three retinoid X receptor (RXRs) isoforms, forming a complex—VD-VDR-RXRs—that binds to vitamin D response elements, directly influencing the transcription of these genes.

After calcitriol, the active molecule of vitamin D, has been synthesized, it dissociates from proteins and, via mechanisms still unknown, penetrates the cytosol of the target cells, binding to the vitamin D receptor. This receptor belongs to the nuclear receptor family of transcription factors present in human cells, which includes a group of proteins that, when activated, are translocated to the cell nucleus, where they interact with DNA and exert their regulatory functions on certain genes. The vitamin D’s binding to VDR induces conformational changes in the vitamin D receptor, leading to its heterodimerization with any of the three retinoid X receptor (RXRs) isoforms—RXRα, RXRβ, and RXRγ—and the posterior translocation of this complex (VD-VDR-RXRs) inside the target cell nucleus. Once inside this organelle, the VD-VDR-RXRs complex binds DNA sequences, called vitamin D response elements, located thousands of base pairs from the codifying sequences of the gene [32], activating or inhibiting the transcription of these genes (Figure 2).

## 3. Current Situation Regarding Serum Levels of Vitamin D

Although calcitrol is the vitamin D metabolite that exhibits biological activity in cells, calcidiol—the VD precursor molecule—has a longer half-life (approximately 15 days); therefore, its serum concentration is over 1000 times higher than that of calcitriol [33]. Because of its high plasma concentration, calcidiol is considered the most important marker of circulating vitamin D levels. Although there are different guidelines for the vitamin D plasma concentration for proper bone health [34,35], the general consensus indicates that serum levels below 12 ng/mL or 30 nmol/L are considered deficient, levels between 12 and 20 ng/mL (30–50 nmol/L) are considered inadequate and levels above 20 ng/mL (50 nmol/L) are considered adequate [36].

As already mentioned in this review, a high proportion of vitamin D reserves in humans is produced by the action of ultraviolet radiation from sunlight. Although prolonged exposure to sunlight is not required, because optimal serum VD concentrations can be achieved with only 5–20 min of exposure per day, there is a high prevalence of hypovitaminosis D worldwide. Recently, Cui et al. [37] conducted a meta-analysis that included 308 studies, involving close to 8 million patients in different countries, in which serum levels of vitamin D were measured; they found that 15.7% of the subjects under study were deficient in this vitamin (<12 ng/mL), and 47.9% had inadequate levels (12–20 ng/mL). In Mexico, Clark et al. [38] determined the plasma concentrations of calcitriol in 585 patients over 14 years of age, reporting adequate levels in only 9.6% of the studied population and deficient levels in 43.6%. The published results of these two investigations reflect the current situation globally; across the world, researchers have found a high prevalence of hypovitaminosis D, even in temperate and subtropical areas. Santos Araujo in Brazil [39], Kamboj in India [40], Martínez-Torres in Colombia [41], and Angeles-Agdepa in the Philippines [42] are just some of the researchers who have demonstrated low levels of serum vitamin D in countries with high exposure to sunlight. Currently, there is no simple explanation for this phenomenon; however, some suggestions can be offered for the high prevalence of this public health problem worldwide, even in countries where there is high exposure to sunlight. These include sedentarism, fear of sunlight exposure as a cause of skin cancer, an increase in everyday activities carried out in closed locations, the use of sunblock, increased levels of obesity, and, finally, an increased incidence of metabolic and malabsorption syndromes among populations [43].

The high prevalence of hypovitaminosis D across the globe, and its possible connection with different extra-skeletal pathologies, must be taken into consideration by the different health-related organizations of the world, as well as by government authorities, so that actions can be jointly implemented to address this public health problem.

## 4. Association of Vitamin D with Influenza and COVID-19

Influenza is an acute respiratory disease caused by the virus of the same name; it affects patients of all ages and has affected humankind for centuries. Influenza virus (InfV) belongs to the *Orthomyxoviridae* family and can be classified into four types: A, B, C, and D, with the first three causing infections in humans and type A being the most likely to cause epidemics. InfV is highly contagious; it is transmitted through the respiratory secretions of infected patients and annually affects a large number of individuals around the world. In the United States alone, between 9 and 45 million new cases are reported every year [44], whereas globally, between 294,000 and 518,000 deaths due to InfV are reported in the same time span [45].

Regarding SARS-CoV-2, at the end of 2019, a series of cases of pneumonia of unknown origin were reported in Wuhan Province, China. A few weeks later, the causal agent of this outbreak was confirmed to be an encapsulated single-stranded RNA virus belonging to the *Coronaviridae* family. Due to its genetic similarity to SARS-CoV-1, this new virus was named SARS-CoV-2, the causative agent of COVID-19. As of 17 December 2023, the World Health Organization (WHO) confirmed the presence of 772 million cases of this infection around the world, with nearly 7 million deaths related to this infection [46].

Due to the presence of the vitamin D receptor on different types of cells of the immune system and its immunomodulatory capacity, several studies have suggested a possible role of circulating vitamin D levels in the prevention of influenza and the development of complications related to this infection [47,48]. Among these studies was a meta-analysis involving 4859 patients, which showed that the administration of VD decreases the risk of influenza (RR = 0.78, 95% CI:0.64–0.95) [20]. Likewise, the GrassrootsHealth study, which included 12,605 participants, showed that individuals with a vitamin D level at or above 60 ng/mL had a 43% lower risk of flu-like illness when compared with individuals whose calcitriol levels were lower than 20 ng/mL (*p* < 0.0001) [49].

At the beginning of the COVID-19 pandemic, considering the existing knowledge of a possible role of vitamin D in the prevention of pro-inflammatory complications in cases of influenza, a relationship between insufficient serum levels of this vitamin and complications arising from this new infection was suggested. In one of the earliest studies that suggested a possible role of vitamin D in the prevention of COVID-19 complications, Lau et al. found a higher incidence of hypovitaminosis D among patients with severe illness admitted to an intensive care unit than among patients with a moderate infections in general wards (84.6% vs. 57.1%, *p* < 0.001) [50]. This work was followed by the study of Ye et al., which found significantly higher rates of calcitriol deficiency in COVID-19 cases (41.9%) when compared to healthy controls (11.1%). Additionally, this study reported that lower serum vitamin D levels were more prevalent in severe/critical cases (80%) than in mild cases (36%). These statistically significant associations remained even after controlling for demographics and comorbidities [51].

Although some studies have suggested that the administration of vitamin D at low doses (800 IU/day) or high doses (3200 IU/day) does not help prevent COVID-19 [52], the opposite has been found in other studies worldwide. In one study carried out in Mexico before the introduction of vaccination against this virus, health-related professionals who cared for hospitalized patients infected with SARS-CoV-2 were included. Of the subjects who finished the study, 94 received 4000 IU/day for a duration of one month, and 98 received a placebo. While only 6.4% of the VD-treated individuals developed the infection, 24.5% in the placebo group were infected (*p* < 0.001) [53]. Furthermore, a recently published meta-analysis, which included 16 different investigations, concluded that vitamin D supplementation has a protective role against COVID-19 (OR = 0.403, 95% IC = 0.218, 0.747) and subsequent admission to an intensive care unit (OR = 0.317, 95% IC = 0.147–0.680) [54].

### 4.1. Effects of Vitamin D on the Prevention of Influenza Virus and SARS-CoV-2 Infections

Research has demonstrated that epithelial respiratory tissue expresses high levels of vitamin D receptor; that these cells can convert calcidiol, the inactive form of the vitamin D, to calcitriol, the active form; and that the calcitriol generated by lung tissue leads to an increase in the expression of VD-regulated genes with key innate immune functions [55]. These observations, coupled with the facts that vitamin D participates in both innate and adaptive responses and that different cells of the immune system express the vitamin D receptor as well as 1α-hydroxylase, suggest that calcitriol could protect the organism against respiratory infections, such as COVID-19 and influenza, in different phases of the infectious process.

Among the many roles that vitamin D might play in protecting against InfV infection and later development of complications, two basic biological aspects must be considered: the anti-infectious attributes of the vitamin and its immunomodulating properties. These two mechanisms are also protective against COVID-19, but in this infection, vitamin D plays an additional protective role through calcitriol’s inhibitory effects on the renin–angiotensin–aldosterone system (RAAS), which is exacerbated during COVID-19 infection and functions as a powerful pro-inflammatory system [56]. Vitamin D inhibits the RAAS by reducing the transcription of the renin gene, leading to decreased renin production by the kidneys. Lower renin levels mean less conversion of angiotensinogen to angiotensin I, which leads to less angiotensin II and aldosterone, molecules that activate the inflammatory cascade [56].

In any infection, the initial mechanisms of the immune response include the production by macrophages of antimicrobial peptides (AMPs), such as cathelicidin, and the activation of Toll-like receptors (TLRs), which are present in macrophages, polymorphonuclear leukocytes, and epithelial cells. One of the functions of AMPs is the disruption of membranes of infectious agents, including InvF and SARS-CoV-2, by forming pores in the membranes of the pathogens. This action damages the viral envelope and can directly inhibit the virus’s ability to survive and replicate. Studies have shown that cathelicidin exhibits a direct lytic activity on InfV [57] and SARS-CoV-2 [58]. Additionally, Wang et al. showed that cathelicidin inhibits SARS-CoV-2 infection via a dual-blockage mechanism. Their in vitro studies provided evidence that this antimicrobial peptide can adhere to the binding domain of the main SARS-CoV-2 receptor, spike 1 protein, and simultaneously to the main host cell receptor, angiotensin-converting enzyme-2 (ACE2), thus decreasing viral adherence and protecting cells against pseudovirion infection [58]. Studies on mice that were infected with either InfV or SARS-CoV-2, and subsequently treated with cathelicidin, confirmed the antiviral activity of this molecule. Animals in the control group presented lower viral loads and less symptomatology for both viruses when compared with the placebo group [57,58]. Regarding TLRs, their role is to function as recognition sensors of invading agents, triggering immune responses, including inflammatory and antiviral actions; it has been shown that VInf is primarily recognized by TLR2, TLR3, TLR4, and TLR9 [59,60], while SARS-CoV-2 by TLR2, TLR4, TLR7, TLR8, and TLR9 [61,62,63]. 

Different studies have shown that vitamin D is a key participant in the activation of both AMPs and TLRs and that this molecule might play a key role in the prevention of viral respiratory infections. During such infections, vitamin D activates the production of cathelicidin LL-37 [64] and may also play an immunomodulating role through the regulation of different TLRs [65]. Lang and Samaras showed that serum cathelicidin concentrations were directly proportional to those of vitamin D, demonstrating that serum calcitrol levels of at least 30 ng/mL are necessary to induce the optimal production of cathelicidin mRNA [66]. In the case of TLRs, animal studies originally showed that calictriol is a strong activator of different TLRs, including TLR2 and TLR5, leading to the up-regulation of AMPs [67]. The demonstration that the initial inflammatory response against InfV and SARS-CoV-2 is strongly mediated by TLRs [68,69] and that vitamin D up-regulates TLR activation of human macrophages [70] suggests that during the initial response against these respiratory viruses, this vitamin plays an important role as an up-regulator of the innate immune response against these agents. 

An additional protective function of vitamin D against influenza virus and SARS-CoV-2 infections stems from its role in the preservation of the pulmonary epithelium’s integrity during these viral infections. Chen et al. demonstrated that calcitriol regulates the expression of proteins such as occludin and claudin, both of which are vital for the maintenance of intimate junctions between cells; this regulation prevents the exposure of cellular receptors to viral adhesion [71], thereby reducing the likelihood of cellular invasion.

### 4.2. Role of Vitamin D in the Prevention of Complications Caused by the Influenza Virus and SARS-CoV-2

The initial phase of InfV or SARS-CoV-2 infection is characterized by the production of type I/III interferons [72]; this is followed by the production of pro-inflammatory cytokines by macrophages, dendritic cells, and infected epithelial cells, whose function is to attract cells and other cytokines to the site of infection, with the objective of eliminating the invading microorganisms and activating the adaptive branch of the host immune response [73]. However, the excessive production of pro-inflammatory cytokines and the lack of a proper modulating mechanism in their expression lead to significant immunopathological damage in the host’s pulmonary tissue, and this can trigger the development of acute respiratory distress syndrome (ARDS), which is the principal complication of infections by these two viruses. Clinically, patients with ARDS present alveolar changes induced by pro-inflammatory cytokines, characterized by thrombosis, necrosis, inflammatory infiltration, and pulmonary edema [74].

Studies performed on patients with ARDS infected with either InfV or SARS-CoV-2 found that these patients presented higher serum pro-inflammatory cytokine concentrations when compared with those of healthy individuals [75,76]. Both groups of patients have been shown to exhibit certain similarities in their cytokine profiles, such as high levels of interleukines 12 (IL-12), 6 (IL-6), 8 (IL-8), 17A (IL-17A), 22 (IL-22), and 23 (IL-23), IFN-γ, tumor necrosis factor-α (TNFα), in both groups, but also marked differences, as interleukines 2 (IL-2) was found to be significantly increased only in influenza cases, while interleukines 4 (IL-4), 7 (IL-7), and 9 (IL-9) were significantly increased in SARS-CoV-2 infections [77].

It has been suggested that the protective role of vitamin D against complications related to InfV and SARS-CoV-2 results from its regulation of the production of pro-inflammatory cytokines. In vitro studies have shown that vitamin D modifies the immune response from a Th1 profile, characterized by the production of pro-inflammatory cytokines, such as INF-γ, IL-2, IL-6, IL-8, and TNF-α, for a Th2 profile, whose primary characteristic is the production of anti-inflammatory cytokines, such as IL-10 and IL-35 [78]. The modulation of IFN-γ expression by vitamin D additionally inhibits the production of reactive oxygen species and nitric oxide, compounds that have been shown in animal studies to actively participate in the pathogenesis of influenza [79]. In vitro studies have also shown that, when pulmonary cells are treated with calictriol prior to influenza H1N1 infection, these cells significantly reduce the production of pro-inflammatory cytokines such as TNFα, IFNβ, IL-8, IL-6, and ISG15 [80]. The regulation of the expression of these cytokines is mainly mediated by the inhibitory effect of vitamin D on the transcription factor NF-kB p65, a molecule that is involved in the control of numerous genes that participate in cellular differentiation, apoptosis, and cytokine production [81], and by the activation of c-maf and GATA-3, the transcription factors involved in the production of Th2-type cytokines [82].

Different studies have shown that additional mechanisms by which vitamin D modulates the inflammatory process include the participation of this molecule in the transformation of dendritic cells to a more tolerogenic state through the induction of fatty acid synthesis [83] and the promotion of macrophage polarization from a pro-inflammatory M1 phenotype to an anti-inflammatory M2 state [84], a phenomenon that reduces the production of pro-inflammatory interleukins. Additionally, vitamin D has been shown to exert an anti-inflammatory effect during viral respiratory infections by increasing the activity of Treg cells, a T lymphocyte subgroup responsible for immune homeostasis and regulation of the inflammatory response. Two demonstrated mechanisms by which these cells inhibit the inflammatory response are (1) through the repression of the activity of CD4+ T cells, B lymphocytes, neutrophils, and dendritic cells [85], and (2) by their own activation in the production of the anti-inflammatory IL-10 [86].

A commonly exhibited feature of patients with ARDS infected with either InfV or SARS-CoV-2 is the presentation of a severe pro-inflammatory response, characterized by the recruitment and further infiltration by neutrophils of the pulmonary tissue with the subsequent expression of neutrophil extracellular traps (NETs), web-like structures released by these cells to trap and kill microorganisms and limit their spread. Although NETs play a key role in the immune response in the elimination of invading agents, these structures might exacerbate the initial inflammatory process and contribute to tissue damage by activating the production of pro-inflammatory cytokines, thereby contributing to the pathogenesis of influenza and COVID-19. Researchers have found that tissue concentrations of NETs in patients infected with either InfV or SARS-CoV-2 who develop ARDS are significantly higher than those in patients with asymptomatic and mild-to-moderate infections [87,88]. Yaqinuddin et al. demonstrated that the excessive production of NETs is directly related to the development of ARDS, as concentrations of pro-inflammatory cytokines increase along with those of NETs, leading to pulmonary damage mediated by these cytokines [89]. Research has also demonstrated that in InfV and SARS-CoV-2 infections, the concentrations of NETs are positively related to APACHE II and MODS severity scores, as increased vascular permeability has been demonstrated in patients with higher concentrations of these structures [87,88].

Studies carried out on patients with systemic lupus erythematosus have demonstrated that the administration of 10 nM of vitamin D reduces the endothelial damage that occurs in such patients because of NETs [90]. This positive interaction is caused by the inhibition of the externalization of these structures and the activation of NADPH oxidase, a key enzyme in their formation [90]. Additionally, Basyreva et al. recently demonstrated in ex vivo studies, using high concentrations of glucose, that the administration of 1000 IU/day of vitamin D for 14 days to healthy individuals decreased the formation of NETs; consequently, VD supplementation was suggested as a preventive measure against the development of type 2 diabetes complications [91]. Based on the studies performed to date on the effects of vitamin D on the inactivation of NETs, the excessive production of these structures in patients infected with InfV and SARS-CoV-2, and the positive outcome of NETs inactivation by this vitamin in other inflammatory diseases, researchers have suggested that the administration of this prohormone to patients infected with these viruses might reduce the complications and mortality caused by these agents [92].

During the initial phases of infections by either the influenza virus or SARS-CoV-2, Toll-like receptors recognize pathogen-associated molecular patterns (PAMPs) related to the viruses, as well as damage-associated molecular patterns (DAMPs) released from injured cells in the lung tissue. These receptors are key participants in the initiation of the inflammatory response; however, as previously described in this review, an exaggerated response of this type, mediated by the excessive activation of TLRs and overproduction of pro-inflammatory cytokines, can lead to lung damage and impaired tissue function, leading to the development of ARDS, the hallmark of severe influenza and SARS-CoV-2 infections. Research has demonstrated that the extracellular nucleoprotein of influenza virus activates the expression of TLR2 and TLR4 and that there is an increased expression of TLR2, TLR3, and TLR9 in human patients infected with H1N1, leading to the production of pro-inflammatory cytokines such as IL-2, IL-6, IFN-γ, and TNF-α, and their overexpression mediates ARDS pathogenesis [60]. Furthermore, using a mouse model, Imai et al. showed that loss of TLR4 expression protects mice from H5N1-induced acute lung injury [93].

Although the exact pathogenesis of SARS-CoV-2 has not been fully elucidated, current knowledge indicates that progression to ARDS is largely influenced by an exaggerated inflammatory response by cytokines such as IL-2, IL-6, and IL-8. New findings have demonstrated that TLRs are vital mediators of COVID-19 immunopathogenesis, as their activation drives the production of pro-inflammatory cytokines. Choudhury et al. found that the spike protein of SARS-CoV-2 strongly binds to the cell surface of TLR4, suggesting a possible role of TLR4 in the pathogenesis of COVID-19 [94]. Following the recognition of the spike protein, TLR2, an additional Toll-like receptor activated during this infection, dimerizes with TLR1 or TLR6, thus initiating a cascade causing a strong inflammatory response that leads to the activation of pro-inflammatory cytokines such as IL-6, IL-1β IL-6, IL-1β, TNFα, CXCL1, CXCL2, and CCL2 [95].

An additional mechanism by which vitamin D modulates the inflammatory cascade in respiratory infections, and by which the chances of developing ARDS are decreased, is by downregulating TLR expression. When human monocytes isolated from peripheral blood were treated with 100 nM of vitamin D, TLR2, TLR4, and TLR9 were downregulated in a time-dependent manner, and as TLR9 expression in monocytes decreased, these cells subsequently secreted less IL-6 in response to TLR9 challenge [96]. Additional research by Sadeghi et al. also showed that vitamin D suppresses the expression of TLR2 and TLR4 protein and mRNA in human monocytes in a time- and dose-dependent fashion, and when treated cells were challenged with bacterial molecules, the TNF-α response was impaired, emphasizing the critical role of TLRs in the induction of inflammation [96]. An additional mechanism by which vitamin D has been suggested to modulate the Toll-like receptor-mediated inflammation cascade is by enhancing a negative feedback mechanism, as calcitrol has been demonstrated to reduce microRNA-155, resulting in an increased production of SOCS1, a potent regulator of cytokines and cell-mediated inflammation, which promotes the negative feedback regulation of TLR signaling [8].

## 5. Association of Vitamin D with Dengue

Dengue is an infection caused by four different types of viruses that are genetically and serologically related (DENV-1, DENV-2, DENV-3, and DENV-4); these viruses belong to the *Flaviviridae* family and are transmitted from person to person through the bites of one of the two Aedes mosquito species, i.e., *A. aegypti* and *A. albopictus*. According to a published report in 2024 by the WHO, over 5 million new cases of dengue are reported every year around the globe, with tropical and subtropical countries being the most affected by this disease [97]. According to one statistical estimate, approximately 14% of these cases occur on the American continent [98], showing a sharp increase in the last decade [97].

According to the clinical presentation of the infected patient, dengue can be classified into four phases: (a) asymptomatic dengue; (b) dengue without warning signs; (c) dengue with warning signs; and (d) severe dengue. The patients who progress to the most severe phase of the infection exhibit greater complications and face a higher risk of death due to multiple organ failure, shock, plasma extravasation, and hemorrhages.

In the last decade, new information has emerged suggesting a possible relationship between dengue symptoms and serum vitamin D levels. In a prospective study conducted in Pakistan, Iqtadar et al. found that a majority (75/97) of hospitalized dengue patients had deficient levels of vitamin D (<12 ng/mL) [99]. Unfortunately, this study did not report the prevalence of hypovitaminosis D in the population where the research was carried out, making it difficult to reach a conclusion regarding the impact of VD deficiency on the development of dengue complications. However, this study did show that patients with a vitamin D serum concentration of ≥20 ng/mL were less likely to develop severe dengue than those with concentrations below this level. The results of a study carried out in Mexico during the 2019 dengue outbreak in the Americas were similar to those described above. In the Mexican study, the authors reported a higher incidence (*p* < 0.05) of dengue without warning signs in patients with serum vitamin D levels below 20 ng/mL [100] than in patients with levels above 30 ng/mL. Additionally, Zaman et al. showed that patients infected with dengue who were treated with high doses of vitamin D at the time of diagnosis showed a lower incidence of severe dengue than those in the placebo group, as only 1.6% (1/62) of patients under VD treatment developed severe dengue and 27.0% (17/62) of the placebo group did develop complications [101]. A major limitation of the three studies described above, in which vitamin D deficiency was linked with the development of symptomatic dengue, is that they were single-center studies with limited numbers of patients included.

The possible link between hypovitaminosis D and the development of symptoms in patients infected with dengue could be explained by two different perspectives: (a) the role played by vitamin D in preventing dengue infection by inhibiting both viral adhesion to the host cells and viral replication and (b) the role played by the vitamin in the modulation of the inflammatory response.

### 5.1. Role of Vitamin D in the Prevention of Dengue Infection

In vitro studies have shown that macrophages that were differentiated in the presence of vitamin D restrict dengue infection by inhibiting viral adhesion to host cells by downregulating mannose receptor expression, one of the key receptors of the virus [102]. Furthermore, different studies have shown that vitamin D not only blocks viral adhesion but also restricts dengue infection by inhibiting virus replication. It has been demonstrated that calcitriol inhibits host cell endoplasmic oxidative stress [103] and the activation of MAPKs, c-Jun N-terminal kinase, and p38 pathways of the macrophage machinery [104], key components required by dengue virions to replicate and infect cells successfully. Therefore, decreased levels of serum calcitriol facilitate dengue infection. Additionally, as previously described for InfV and SARS-CoV-2 infections, vitamin D is an important activator of the antimicrobial peptide cathelicidin, which has been shown to inhibit dengue virus infectivity and replication by binding to the viral envelope protein [105].

### 5.2. Role of Vitamin D in the Prevention of Complications Caused by Dengue

To date, the exact mechanisms by which an infected patient progresses to severe dengue are still unknown; however, all such patients present one common feature: an exaggerated inflammatory response, characterized by the elevated serum concentrations of pro-inflammatory cytokines such as IL-6, IL-8, TNF-α, CXCL10, CXCL11 and RANTES [106]. Additionally, Sánchez-Vargas et al. showed that patients with severe dengue also excessively produce IL-17A [107], a pro-inflammatory cytokine produced by Th17 cells, a subset of CD4+ T helper cells; an exaggerated expression of IL-17A correlates with dengue complications caused by activating neutrophils and inducing the release of inflammatory mediators from these cells. Furthermore, research has shown that an excessive level of IL-6 cytokine produced following viral infection promotes the development of IL-17A-producing pathogenic helper T cells, and that IL-6, together with IL-17, synergistically promotes viral survival by inhibiting cellular apoptosis and cytotoxic T cell function [108]. In addition to the overexpression of the pro-inflammatory cytokines mentioned above, Guabiraba et al. demonstrated using an experimental animal model with a mouse-adapted DENV serotype 2 strain that, upon infection, mice also hyper-secrete IL-22, a pro-inflammatory cytokine that is secreted by Th17 lymphocytes and that modulates the production of IL-17A [109]. Different in vitro studies have shown that the binding between dengue virus—specifically, the viral antigens NS5 and NS4—and host cells leads to the activation of the production of these pro-inflammatory cytokines [110,111]. All these molecules that are overexpressed during severe dengue have been shown to increase the permeability of the endothelium and act as chemoattractants for other components of the immune response, contributing to the development of an exaggerated inflammatory response, which favors plasmatic extravasation [110,111], a hallmark of dengue’s complications.

Vitamin D not only protects against viral adhesion and replication in dengue, but it also simultaneously plays a key role as an immunomodulator of the inflammatory response, a key step in the prevention of severe dengue. In an in vitro study using dengue-infected monocytes, Puerta-Guardo et al. demonstrated that vitamin D significantly reduced the levels of TNF-α, IL-6, IL-12p70, and IL-1β, all pro-inflammatory cytokines, produced by infected cells [112]. Additionally, Giraldo et al. demonstrated that monocyte-derived macrophages obtained from healthy individuals who received 4000 IU/day of vitamin D were less susceptible to dengue virus invasion and produced fewer pro-inflammatory cytokines and more anti-inflammatory ones than cells obtained from donors who were treated with only 1000 IU/day [18].

Research has demonstrated that vitamin D could reduce the likelihood of developing severe dengue by modulating the cytokine-mediated inflammatory response through different mechanisms, including the downregulation of pro-inflammatory cytokine production by Th17 cells. Studies in mice and in vitro have demonstrated that, in a VDR-dependent manner, vitamin D inhibits the production of these cytokines by this subset of cells [113]. Furthermore, research has shown that calcitriol modulates the expression of IL-22 by Th17 cells through the inhibition of *il22* gene transcription [114], and a reduced expression further results in a decreased production of pro-inflammatory IL-17. Martínez-Moreno et al. also demonstrated that monocyte-derived dendritic cells obtained from healthy volunteers treated with 4000 IU of VD showed a decreased mRNA expression of TLR3, 7, and 9, the downregulation of pro-inflammatory cytokines IL-12/IL-8, and an increased anti-inflammatory IL-10 secretion in response to DENV-2 infection [115]. In summary, the excessive production of pro-inflammatory cytokines in severe dengue causes vascular dysfunction, specifically increased vascular permeability, leading to plasma leakage. Research has shown that vitamin D might modulate the expression of these molecules; thus, calcitriol may be a potential anti-dengue compound. However, clinical trials in humans are still needed to conclusively demonstrate the positive effects of vitamin D in preventing the development of severe dengue.

An additional mechanism through which the active form of VD modulates the immune response is the regulation of the production of miRNAs, small non-coding RNA molecules that regulate gene expression at the post-transcriptional level. One of the main mechanisms involved in the multiorgan failure observed in patients with severe dengue is the excessive production of TNF-α, which in high concentrations favors plasma extravasation [116]. Castillo et al. demonstrated that vitamin D could reduce the risk of dengue complications by regulating the production of TNF-α by inhibiting the expression of several miRNAs involved in its regulation (miR-182-5p, miR-130a-3p, miR-125b-5p, and miR-155-5p) [117]. Furthermore, the same researchers demonstrated a possible additional protective mechanism of vitamin D in dengue virus infection, by the induction of the overexpression of other miRNAs (miR-182-5p, miR-130a-3p, miR-125b-5p, and miR-155-5p), which decrease the replication rates of the virus in macrophages [117].

Finally, another component of the immune response that seemed to be involved in the pathogenesis of dengue is the participation of CD4^+^ regulatory T cells, Treg cells, during the infectious process. Although regulatory T-cells are thought to be properly expanded in dengue, research has demonstrated that Treg cells in patients with severe dengue have less suppressive capacity compared to those obtained from healthy donors and present a Th1-like phenotype, characterized by the secretion of pro-inflammatory cytokines, the major contributors of the detrimental effects seen in severe dengue cases [118]. Additionally, the expression of chemokine receptor CCR4 is low in most of these cells in severe dengue, thus reducing their migration to sites of inflammation, and leading to an enhanced immune response [119].

Patients infected with dengue could benefit from vitamin D supplementation to modulate their pro-inflammatory response, as it has been shown that a 12-week high-dose oral cholecalciferol supplementation (140,000 IU/month) in healthy volunteers increases peripheral CD4⁺ Tregs without negatively affecting the frequency of other immune cells [120]. As previously described in this review, vitamin D modulates Th17 activation, downregulating the pro-inflammatory cytokine response. In vivo and in vitro studies have demonstrated that calcitriol treatment reduces Th17 activity while promoting Treg cell activation [121].

## 6. Future Research

Despite the substantial scientific evidence supporting the role of vitamin D in the activation and regulation of the immune system and its potential protective effects against infections such as influenza, COVID-19, dengue, and their exaggerated inflammation-mediated complications, there remain critical gaps that future research should address. In this final section of this review, we include some points on the relationship between vitamin D and the immune system to be considered by future studies.

### 6.1. Definition of Vitamin D Reference Values for a Healthy Immune System

The current guidelines on the levels of vitamin D have been established to achieve a healthy skeletal system; however, there is a shortage of information on the right amount of this vitamin to maintain an equilibrium between an appropriate inflammatory/anti-inflammatory ratio to fight infections. Future research should aim to establish standardized guidelines, particularly in high-risk populations, to determine the optimal dosage for preventing infections and reducing complications. In addition, clinical trials will need to determine the interaction of vitamin D with other micronutrients and its role in the epigenetic regulation of the immune response against influenza, COVID-19, and dengue.

### 6.2. Role of Vitamin D in Emerging Infectious Diseases

Considering the immunomodulatory properties of vitamin D, future research should explore its potential role in newly emerging or re-emerging infectious diseases. Investigating the impact of calcitrol on other viral, bacterial, or fungal infections could expand its clinical applications beyond the infections covered in this review.

### 6.3. Long-Term Effects of Vitamin D Deficiency on Immune Function

While many studies on the impact of vitamin D on infections focus on the acute phase of diseases, there is limited information on the impact of VD deficiency on the immune system over time. Longitudinal studies could help determine whether persistent deficiency increases susceptibility to recurrent infections or long-term immune dysregulation.

### 6.4. Effect of Vitamin D on Vaccines

As demonstrated in this review, vitamin D plays a multifunctional role in the infection process, upregulating certain cells and molecules and downregulating others. Future clinical trials should take this property of calcitrol into consideration during the development of new vaccines against infectious agents and test whether the supplementation of VD, before or after vaccination, improves immune responses or reduces adverse effects.

Future studies could also investigate whether there is a range of vitamin D levels that enhances the effectiveness of vaccines, especially in high-risk populations, including those living in areas with limited sunlight exposure.

## 7. Conclusions

Dengue and influenza viruses, for many years, and COVID-19, since 2020, have caused high incidences of morbidity and mortality in humans. Despite belonging to different viral families, these microorganisms exhibit a common trait: their capacity to cause complications through their cytopathic effect on target tissue and through the excessive inflammatory response produced by the human host to the infection. In this review, we describe the main immunological mechanisms that are activated during the infections caused by these viruses and those that lead to the development of their complications. The different biological processes by which vitamin D might prevent these infections are also described; these include the activation of antiviral molecules (cathelicidins) and endothelial protective molecules (occludin and claudin), as well as other molecules involved in the activation of the immune response (TLRs and miRNAs). This review also describes the protective role of vitamin D in avoiding complications of influenza, SARS-CoV-2, and dengue infections. This protection is achieved through the immunomodulatory capacity of this vitamin, which inhibits the exaggerated inflammatory response, a hallmark of these infections. The switch from a pro-inflammatory state to one of decreased inflammation is achieved through a series of mechanisms that involve the inactivation of a complex network of immune molecules and cells, such as Toll-like receptors, pro-inflammatory cytokines (IL-2, IL-6, IL-8, IL-22 and TNF) and immune structures (NETs), and the activation of another network, including regulatory Treg cells and anti-inflammatory cytokines (IL-10).

Despite the overwhelming evidence that vitamin D plays an important role in preventing the aforementioned infections and their complications, there are still several unanswered questions about the interaction of this molecule with the immune system. Future research in this field should result in a deeper understanding of the role that vitamin D plays in controlling immunological processes, thereby creating new treatment and prevention opportunities in the field of infectious diseases.

## Data Availability

Not applicable.
